# Identification of novel genetic risk factors of dilated cardiomyopathy: from canine to human

**DOI:** 10.1186/s13073-023-01221-3

**Published:** 2023-09-18

**Authors:** Julia E. Niskanen, Åsa Ohlsson, Ingrid Ljungvall, Michaela Drögemüller, Robert F. Ernst, Dennis Dooijes, Hanneke W. M. van Deutekom, J. Peter van Tintelen, Christian J. B. Snijders Blok, Marion van Vugt, Jessica van Setten, Folkert W. Asselbergs, Aleksandra Domanjko Petrič, Milla Salonen, Sruthi Hundi, Matthias Hörtenhuber, DoGA consortium, Carsten Daub, César L. Araujo, Ileana B. Quintero, Kaisa Kyöstilä, Maria Kaukonen, Meharji Arumilli, Riika Sarviaho, Jenni Puurunen, Sini Sulkama, Sini Karjalainen, Antti Sukura, Pernilla Syrjä, Niina Airas, Henna Pekkarinen, Ilona Kareinen, Hanna-Maaria Javela, Anna Knuuttila, Heli Nordgren, Karoliina Hagner, Tarja Pääkkönen, Antti Iivanainen, Kaarel Krjutskov, Sini Ezer, Auli Saarinen, Shintaro Katayama, Masahito Yoshihara, Abdul Kadir Mukarram, Rasha Fahad Aljelaify, Fiona Ross, Amitha Raman, Irene Stevens, Oleg Gusev, Danika Bannasch, Jeffrey J. Schoenebeck, Juha Kere, W. Glen Pyle, Jonas Donner, Alex V. Postma, Tosso Leeb, Göran Andersson, Marjo K. Hytönen, Jens Häggström, Maria Wiberg, Jana Friederich, Jenny Eberhard, Magdalena Harakalova, Frank G. van Steenbeek, Gerhard Wess, Hannes Lohi

**Affiliations:** 1 Department of Medical and Clinical Genetics, University of Helsinki, Haartmaninkatu 8, 00290 Helsinki, Finland; 2 Department of Veterinary Biosciences, University of Helsinki, Agnes Sjöbergin katu 2, 00790 Helsinki, Finland; 3 Folkhälsan Research Center, Haartmaninkatu 8, P.O.Box 63, 00290 Helsinki, Finland; 4 Department of Animal Breeding and Genetics, Swedish University of Agricultural Sciences, Uppsala, Sweden; 5 Department of Clinical Sciences, Swedish University of Agricultural Sciences, Uppsala, Sweden; 6 Institute of Genetics, Vetsuisse Faculty, University of Bern, Bern, 3001 Switzerland; 7 Department of Genetics, University Medical Centre Utrecht, Utrecht University, Utrecht, The Netherlands; 8 Department of Cardiology, Division Heart & Lungs, University Medical Center Utrecht (UMCU), Utrecht, The Netherlands; 9 Regenerative Medicine Centre Utrecht, University of Utrecht, Utrecht, The Netherlands; 10 Amsterdam University Medical Centers, Department of Cardiology, University of Amsterdam, Amsterdam, The Netherlands; 11 Health Data Research UK and Institute of Health Informatics, University College London, London, UK; 12 Small Animal Clinic, Veterinary Faculty, University of Ljubljana, Ljubljana, 1000 Slovenia; 13 Department of Biosciences and Nutrition, Karolinska Institutet, Huddinge, Sweden; 14 Research Programs Unit, Stem Cells and Metabolism Research Program, University of Helsinki, Helsinki, Finland; 15 Department of Biomedical Sciences, University of Guelph, Guelph, ON Canada; 16 IMPART Investigator Team Canada, Dalhousie Medicine, Saint John, NB Canada; 17 Wisdom Panel Research Team, Wisdom Panel, Kinship, Helsinki, Finland; 18 Department of Human Genetics, Amsterdam University Medical Center, Amsterdam, The Netherlands; 19 Department of Medical Biology, Amsterdam University Medical Center, Amsterdam, The Netherlands; 20 Department of Equine and Small Animal Medicine, University of Helsinki, Helsinki, Finland; 21 LMU Small Animal Clinic, Ludwig Maximilians University of Munich, Munich, Germany; 22 Department of Clinical Sciences, Faculty of Veterinary Medicine, Utrecht University, Yalelaan 108, Utrecht, 3584 CM The Netherlands

**Keywords:** Cardiac, Cardiology, Genetics, Companion animal, Arrhythmia, GWAS, Complex trait, Transcriptomics

## Abstract

**Background:**

Dilated cardiomyopathy (DCM) is a life-threatening heart disease and a common cause of heart failure due to systolic dysfunction and subsequent left or biventricular dilatation. A significant number of cases have a genetic etiology; however, as a complex disease, the exact genetic risk factors are largely unknown, and many patients remain without a molecular diagnosis.

**Methods:**

We performed GWAS followed by whole-genome, transcriptome, and immunohistochemical analyses in a spontaneously occurring canine model of DCM. Canine gene discovery was followed up in three human DCM cohorts.

**Results:**

Our results revealed two independent additive loci associated with the typical DCM phenotype comprising left ventricular systolic dysfunction and dilatation. We highlight two novel candidate genes, *RNF207* and *PRKAA2*, known for their involvement in cardiac action potentials, energy homeostasis, and morphology. We further illustrate the distinct genetic etiologies underlying the typical DCM phenotype and ventricular premature contractions. Finally, we followed up on the canine discoveries in human DCM patients and discovered candidate variants in our two novel genes.

**Conclusions:**

Collectively, our study yields insight into the molecular pathophysiology of DCM and provides a large animal model for preclinical studies.

**Supplementary Information:**

The online version contains supplementary material available at 10.1186/s13073-023-01221-3.

## Background

Dilated cardiomyopathy (DCM) is a severe cardiac muscle disorder affecting both humans and dogs. An important subset of human DCM patients have a genetic background and follow an autosomal dominant mode of inheritance with reduced penetrance [1]. In humans, over a hundred putative risk genes have been identified that encode proteins involved in many essential functions and structures of the heart, of which nineteen have moderate to solid evidence for a causative gene-disease relationship [2]. Several genome-wide association studies (GWASs) [3–6] have been performed using non-familial DCM cases resulting in associated variants. Despite these extensive efforts in genetic diagnosis, many affected patients remain without a molecular diagnosis [1].

DCM is the second most common heart disease in dogs [7–11], and these patients are referred to specialized clinics. Dogs with DCM form both a valuable non-human patient cohort and an essential natural model organism for human DCM with similar disease etiology and progression, clinical signs, sex predisposition, prognosis, and response to medical treatment strategies [12, 13]. Many medium- to large-sized dog breeds have an increased genetic predisposition to develop DCM, including Dobermanns, Irish Wolfhounds, Newfoundland dogs, and Great Danes [14–17]. The cumulative prevalence of DCM specifically in Dobermanns is strikingly high (over 1:2) [18] in contrast to human DCM (1:250–400 [19] up to 1:2500 [20]). The morphological and clinical aspects of DCM in Dobermanns are well characterized: the typical DCM phenotype is left ventricular systolic dysfunction with secondary left ventricular enlargement, which often follows an arrhythmogenic phase in which the affected dogs have ventricular premature complexes (VPCs), and the disease is progressive with a typical onset in middle-aged to old dogs [14, 18, 21]. Additionally, sex affects the manifestation: females are more likely to have VPCs as a sole abnormality even in old age, whereas males tend to show echocardiographic changes earlier than females [18].

In the last couple of years, purebred dogs have been the subject of a public debate focusing on their health and welfare. As a result of 200 years of inbreeding and selection for specific phenotypic characteristics, the dog has become a magnifying glass for genetic disorders [22, 23]. The canine genome exhibits more homology to the human genome than rodents [24], typically used in DCM modeling [25]. Intrabreed mapping approaches resemble population-specific GWASs in human cohorts, and the high breed-specific prevalence of disorders, genetic homogeneity, and extensive linkage disequilibrium enable remarkably well-powered studies in Mendelian diseases [24, 26–31]. However, previous attempts to dissect the complex genetic background of DCM in Dobermanns have been limited by inadequate sample sizes or inconclusive functional follow-up, leading to contradictive findings and thus warranting the need for a more extensive and comprehensive study [32–40].

We present a comprehensive multi-omics study conducted in a spontaneous canine model of DCM. With the largest cohort assembled to date, comprising DCM-affected and healthy Dobermanns, our research aims to unravel the genetic factors and underlying mechanisms contributing to DCM. Through a combination of genome-wide association studies (GWAS), whole-genome sequencing, transcriptome analysis, and immunohistochemistry, we investigate the genetic landscape of DCM in this model. Furthermore, we extend our canine gene discovery to three human DCM cohorts, establishing potential translational implications. The identification of genetic DCM risk factors through our study holds promise for advancing DCM diagnostics across species, including the development of a prospective genetic test for veterinary medicine and dog breeding.

## Methods

### Clinical study cohorts and classification criteria

We included 540 privately owned Dobermanns between 1999 and 2019 from several facilities in Europe: 400 dogs at Ludwig Maximilian University, Munich, Germany, and 31 dogs at the University Small Animal Hospital, University of Helsinki, Helsinki, Finland (“main cohort”); 65 dogs at Utrecht University, Utrecht, the Netherlands, and 9 dogs at the University Small Animal Clinic, University of Ljubljana, Ljubljana, Slovenia (“Utrecht cohort”); and 35 dogs at the Swedish University of Agricultural Sciences (SLU), Uppsala, Sweden (“Uppsala cohort”) (Additional file 2: Table S1). Clinical examinations consisted of a physical examination and a standard echocardiographic examination, including an assessment of left ventricular size by either Simpson’s method of disks (SMOD) or M-mode and, for all dogs of the main cohort, a 24-h Holter monitoring. The cutoff values, presented in Table 1, were based on the recommendations of Wess et al. [21, 41–43].

**Table 1:** Measurements and cutoff values for classifying the examined Dobermanns into groups with normal or abnormal echocardiography and 24-h ECG (Holter) results. The cutoffs were based on the recommendations of Wess et al. [12]

Measurement	Normal	Abnormal
LVEDV/BSA (SMOD)	< 95 ml/m^2^	≥ 100 ml/m^2^
LVESV/BSA (SMOD)	< 55 ml/m^2^	≥ 60 ml/m^2^
LVIDd (M-mode)	< 46 mm (female)	≥ 46 mm (female)
	< 48 mm (male)	≥ 48 mm (male)
LVIDs (M-mode)	< 36 mm (female and male)	≥ 36 mm (female and male)
24-h Holter monitoring	< 50 VPCs/24 h	≥ 300 VPCs/24 h or 50–300 VPCs/24 h in two subsequent records within 1 year

*BSA* body surface area, *LVEDV* left ventricular end-diastolic volume, *LVESV* left ventricular end-systolic volume, *LVIDd* left ventricular internal diameter end diastole, *LVIDs* left ventricular internal diameter end-systole, *SMOD* Simpson’s method of disks, *VPC* ventricular premature complex

From the measured values, fractional shortening (FS) was calculated from left ventricular internal end-diastolic and end-systolic dimensions ((LVIDd − LVIDs)/LVIDd × 100) and left atrial to aortic root diameter ratio (LA/Ao) from the diameters of the left atrial appendage and the aortic root. Congestive heart failure (CHF) was confirmed by thoracic radiographs showing pulmonary edema as indicated by an interstitial or alveolar lung pattern in the caudo-dorsal part of the lung, plus enlargement of the left atrium and cardiomegaly.

We classified the dogs with detailed clinical information (*N* = 431, from Finland and Germany—“main cohort,” Table 2) into several subcohorts based on the findings as follows: (i) “echo only” (*N* = 45): dogs with an abnormal echocardiography result, a normal Holter result, and no CHF; (ii) “echo + arrhythmia” (*N* = 113): dogs with an abnormal result for both echocardiographic examination and Holter monitoring and no CHF; (iii) “arrhythmia only” (*N* = 70): dogs with a normal echocardiography result, an abnormal Holter result, and no CHF; (iv) “CHF” (*N* = 55): dogs at the overt stage of DCM; and (v) “healthy” (*N* = 148): dogs with a normal result for both echocardiographic examination and Holter monitoring that were at least 6 years old at the time of the examination.

**Table 2:** Characteristics of the three canine cohorts and their role in the genetic association study

**Cohort**	**Country of origin**	**Number**	**Purpose**	**Genotyping**
Main cohort (*n* = 431)	Germany	400	Discovery	Axiom canine genotyping array
	Finland	31		
Utrecht cohort (*n* = 74)	The Netherlands	65	Replication	
	Slovenia	9		
Uppsala cohort (*n* = 35)	Sweden	35	Validation	Sanger sequencing
	*Total*	*540*		

To enrich for dogs with a very high likelihood of DCM phenotype, we used cutoff values of SMOD EDVI ≥ 100 ml/m^2^ and ESVI ≥ 60 ml/m^2^, which are slightly higher than the recommended values [21]. For the control group (“healthy”), we used the recommended SMOD EDVI < 95 ml/m^2^ and ESVI < 55 ml/m^2^ values. Dogs in the “gray zone” or when only one of the parameters was abnormal were excluded from this study. Only dogs fulfilling the Holter criteria clearly were included, and dogs in gray zones were excluded.

Dogs that had supraventricular premature contractions were included, but they were only put into the arrhythmia group if there were also ventricular premature contractions detected according to the criteria stated before. According to a recent study, dogs with only supraventricular premature contractions without VPCs were classified according to the ECHO criteria and not counted as arrhythmias, because supraventricular premature contractions were not early markers of DCM [44]. Dogs with atrial fibrillation were included in the arrhythmia groups and the respective ECHO classification because all dogs with atrial fibrillation also had VPCs on Holter examinations.

In addition to the 431 dogs of the main cohort included in GWAS, we evaluated a cohort of 74 dogs examined at Utrecht University, Utrecht, the Netherlands, and Small Animal Clinic, University of Ljubljana, Ljubljana, Slovenia (“Utrecht cohort”). These dogs were not included in the main cohort due to the lack of comprehensive phenotypic information. Clinical parameters collected include LVIDd, LVIDs, FS, and LA/Ao. Dogs were confirmed as unaffected when older than 9 years old and without clinical findings or signs indicative of DCM.

To enable genetic analyses, we collected EDTA blood samples from the study cohorts with the owners’ informed consent and stored them at −20 °C or −80 °C until genomic DNA was extracted. Extraction was performed with either a semi-automated Chemagen extraction robot (PerkinElmer Chemagen Technologie, Waltham, MA, USA), a Maxwell RSC 48 instrument with the Maxwell RSC Whole Blood DNA Kit (Promega, Madison, WI, USA), a Nucleon BACC2 kit (GE Healthcare, Chicago, IL, USA), or the QiaSymphony platform using the Mini-prep kit (Qiagen, Hilden, Germany). DNA concentration was measured with NanoDrop ND-UV/Vis Spectrophotometer or Denovix DS-11 Spectrophotometer. The samples were collected under the permission of the animal ethical committee of the County Administrative Board of Southern Finland (ESAVI/6054/04.10.03/2012, ESAVI/343/04.10.07/2016, and ESAVI/25696/2020); the ethical committee of the Center of Clinical Medicine, Munich, LMU University (60-18-11-2015); and the ethical committee of the Swedish Board of Agriculture (No. C2/12 (2012-02-24) and No. C12/15 (2015-02-27)). All sampling procedures in clinics conformed to the guidelines from Directive 2010/63/EU of the European Parliament on the protection of animals.

### Association analyses

To identify new DCM loci, we performed genome-wide array SNP genotyping of genomic DNA prepared from blood samples taken from 431 clinically examined Dobermanns in the “main cohort” in three batches with the Axiom Canine Genotyping Array Sets A and B (1,269,218‬ markers) (Thermo Fisher Scientific, Waltham, MA, USA). Quality control (QC) was performed with PLINK v1.9 [45]. Each batch was pruned for a genotyping rate of > 95% per marker and individual before merging. Pre-analytical QC was conducted separately for each analysis and included genotyping rate of > 95% per marker and individual as well as a minor allele frequency of > 5% and Hardy-Weinberg test score of > 1 × 10^−8^. Three samples that failed the X-chromosomal sex check were discarded. Only markers classified into PolyHighResolution, NoMinorHom, or MonoHighResolution categories in every batch were retained. Finally, only one marker at each duplicate position was retained, and the rest were excluded from the analysis.

We analyzed the data with univariate linear mixed model association with a likelihood ratio test implemented in GEMMA (version 0.98.1) [46] with sex as a covariate to account for the sex bias in DCM [18] and a genetic relatedness matrix to correct for population stratification. Multiple testing correction was implemented with both Bonferroni correction and by correcting with the effective number of independent tests (*M*_eff_) estimated with simpleM [47–49]. We used the LD-based simpleM method as the primary correction approach, as Bonferroni correction is overly conservative for data with substantial LD. Finally, post-analytical QC included evaluation of population stratification from multidimensional scaling (MDS) plots and quantile-quantile (Q-Q) plots and assessment of SNP correlation structure calculated with PLINK [45].

### Odds ratios and interaction analysis

To explore the genome-wide significant loci discovered in the association analyses, we examined the association and interaction of the risk SNPs chr5:60,531,090 and chr5:53,109,178 with left ventricular systolic dysfunction and dilatation using logistic regression, with 213 cases from the “echo only,” “echo + arrhythmia,” and “CHF” subcohorts as events and 148 controls from the “healthy” subcohort as non-events. Of these dogs, 52% were male and 48% female. Due to technical genotyping errors, the genotype for chr5:60,531,090 was missing for two dogs and chr5:53,109,178 for one dog, and thus, the final number of dogs included in the analysis was 358. We explained the echocardiographic phenotype with genotypes at chr5:60,531,090 and chr5:53,109,178 and the dog’s sex.

We carefully assessed model fit. First, we evaluated and plotted influential data points with packages “broom” [50], “dplyr” [51], and “ggplot2” [52] and did not find any outliers. Second, we evaluated multicollinearity with the package “car” [53]. The generalized variance inflation factor estimate was less than 1.05 for all variables, indicating no multicollinearity. Third, we calculated the area under the receiver operating characteristic curve (AUC) to evaluate how well the model is able to classify cases and controls. AUC was 0.789, indicating good classification.

After fitting the model, we estimated the overall effect of the explanatory variables with analysis of variance (ANOVA) with the package “car” [53]. Furthermore, we calculated the estimated marginal means for all explanatory variables using the package “emmeans” [54] to obtain means for variable levels and evaluate their pairwise differences.

The significance cutoff value was *P* < 0.05. Logistic regression as well as assessment of model fit and evaluation of the results were conducted in R (version 3.6.2) [55].

### Whole-genome sequence analysis

**Fig. 1:**
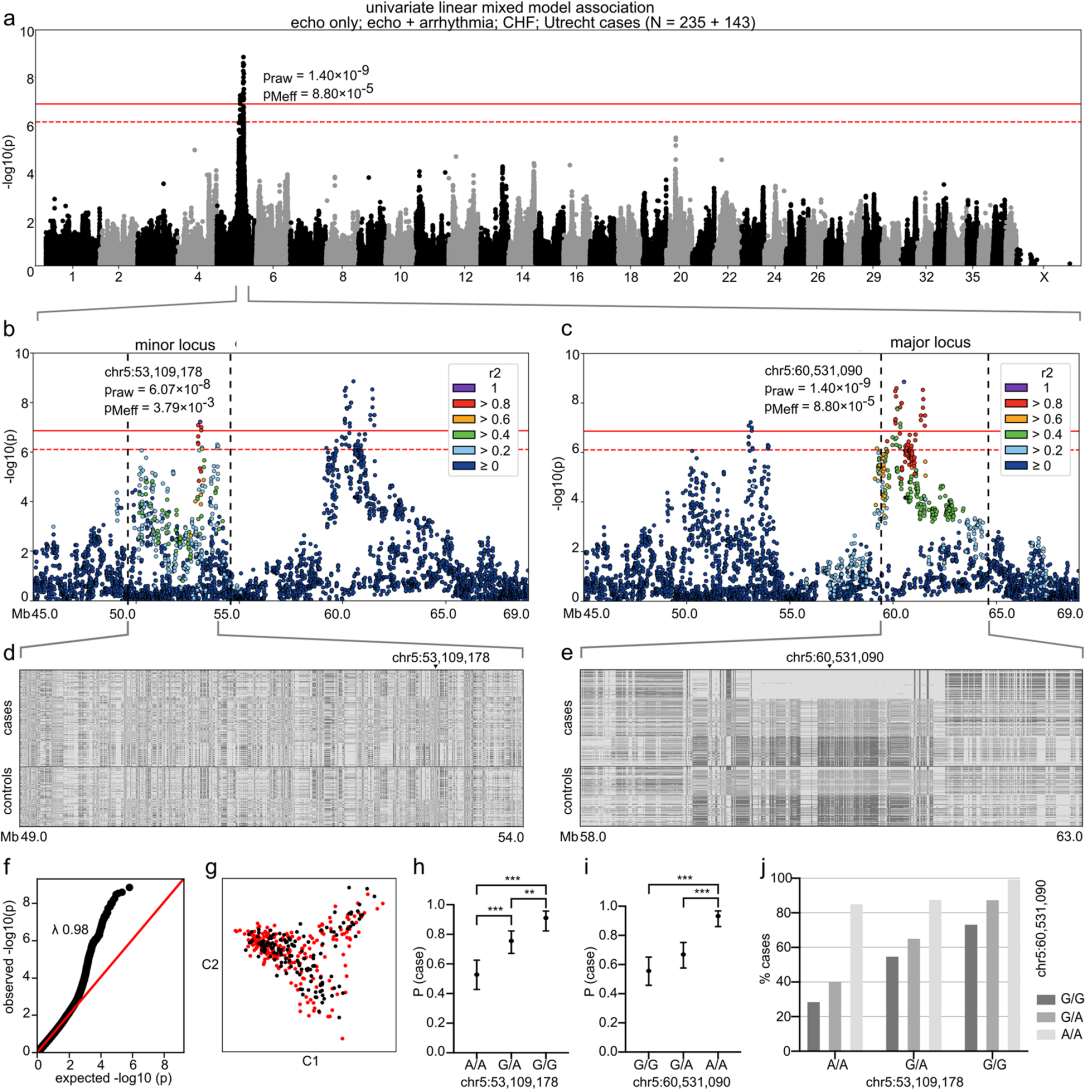
GWAS with a univariate linear mixed model. The analysis included 235 cases from the “echo only,” “echo + arrhythmia,” and “CHF” subcohorts and Utrecht cohort and 143 controls from the “healthy” subcohort. In **a**–**c**, Bonferroni-corrected and Meff significance thresholds are indicated with solid and dashed red lines, respectively. In **d** and **e**, the colors indicate the homozygous genotype for the case major allele (light gray), heterozygous genotype (middle gray), and homozygous genotype for the case minor allele (dark gray). In **h** and **i**, error bars indicate 95% confidence limits and asterisks ** and *** *p*-values < 0.01 and < 0.001, respectively. **a** A genome-wide significant two-locus signal occurs on chromosome 5: the major locus resides at 60 Mb and the minor locus at 53 Mb. The most significant SNP (*p*_raw_ = 1.40 × 10^–9^, *p*_Meff_ = 8.80 × 10^–5^) is located at chr5:60,531,090. **b** A locus plot of chr5:45.0–69.0 Mb and SNP correlation structure of the index SNP chr5:53,109,178. **c** A locus plot of chr5:45.0–69.0 Mb and SNP correlation structure of the index SNP chr5:60,531,090. **d** Genotype plot of chr5:59–54 Mb. **e** Genotype plot of chr5:58–63 Mb. **f** Q-Q plot of the *p*-values (likelihood ratio test). **g** A multi-dimensional scaling (MDS) plot of the cases (red) and controls (black). **h** Probability of case status (P (case)) by genotype at chr5:53,109,178 (*N* = 372). **i** Probability of case status (P (case)) by genotype at chr5:60,531,090 (*N* = 372). **j** Frequency of case status by joint genotypes at ch5:53,109,178 and chr5:60,531,090 (*N* = 372)

We performed whole-genome sequencing of genomic DNA prepared from blood samples from twelve affected and one unaffected Dobermann using the Illumina HiSeq X ultra-high-throughput sequencing platform (Illumina Inc., San Diego, CA, USA) with 30 × target coverage (paired-end reads, 2 × 150 bp) (Novogene Bioinformatics Institute, Beijing, China). We originally sequenced ten dogs, including five from the “echo only” subcohort and five from the “echo + arrhythmia” subcohort in the Finnish and German populations, for our WGS analysis. Samples representing opposite homozygous haplotypes (Fig. 1e) were selected to discover possible candidate variants in the associated major locus in different clinical subgroups. Later during the study, three dogs from the Uppsala cohort, including one dog from the “echo only” subcohort, one dog from the “CHF” subcohort, and one dog from the “healthy” subcohort, were sequenced for an auxiliary analysis of a candidate gene; they were not included in the full WGS analysis. In addition, we used variant data from 393 whole-genome sequences from wolves and 87 non-Dobermann breeds that were either publicly available from the Dog Biomedical Variant Database Consortium (DBVDC) [56] or sequenced for our other studies (Additional file 2: Table S2).

For our in-house data, mapping, variant calling, and annotation for single nucleotide variants (SNV), small insertions/deletions (indel), structural variants (SV), and mobile element insertions (MEI) were performed as previously described using canFam3.1 as the reference genome [57, 58]. To account for inaccuracies in indel calling, genotypes of indel variants were considered heterozygous and homozygous if an individual had one or two copies of any non-reference allele, respectively. Additional annotations were obtained from the catalogs of Gene Ontology (GO) [59, 60], Mouse Genome Informatics (MGI) [61], and Human Protein Atlas [62]. The impact of exonic variants was predicted with SIFT [63].

To identify the variants that tag the risk alleles at chr5:60,531,090 and chr5:53,308,774, we retrieved all SNVs, indels, SVs, and MEIs in the regions in the ten initially sequenced affected dogs. In the chr5:60 Mb locus, we required that cases with one or two risk alleles shared the variants in heterozygous or homozygous state. In the chr5:53 Mb locus, we analyzed the two groups of cases separately according to genotype at chr5:53,109,178 (G/G or A/A), as based on allele frequency it could not be determined whether the G allele was introduced in the Dobermann breed formation resulting in an increased risk of echocardiographic changes or if the A allele was introduced resulting in a protective effect (Additional file 2: Table S3). In the filtering step, we omitted breeds with a known high prevalence of cardiac disease and utilized 366 control genomes with SNVs/indels and 211 with SVs/MEIs. As DCM occurs across dog breeds, we allowed a maximum of 10% of controls to carry the alternate allele in the heterozygous or homozygous state.

### Sanger sequencing

To screen a candidate variant at chr5:60,111,983, we genotyped it in 35 dogs from the Uppsala cohort with standard PCR and Sanger sequencing. Using Primer3 [64], the following primers were designed: 5′-TCTCCCTCTTCCTCTCACCA-3′ (forward) and 5′-TGTCTCCCATGATCTCGGC-3′ (reverse). The PCR products were sequenced with an Applied Biosystems ABI3730XL DNA Analyzer capillary sequencer (Thermo Fisher Scientific) at the Institute for Molecular Medicine Finland (FIMM) core facility. The sequencing results were analyzed with UGENE (version 1.32.0) [65]. Finally, the association of the variant to left ventricular systolic dysfunction and dilatation was assessed with the one-tailed chi-squared test assuming the A allele to be associated.

### RNA samples

We performed RNA sequencing on cardiac tissue to evaluate the differences in RNA expression between DCM-affected and unaffected Dobermanns. Following signed informed owner consent, we collected the tissues from four healthy and five affected Dobermanns (“Uppsala RNA cohort,” a subset of the Uppsala cohort) euthanized for reasons unrelated to this study at the University Animal Teaching Hospital in association with SLU, Uppsala, Sweden. Euthanization was initiated by intravenous injection in the cephalic vein of propofol (2,6 diisopropylfenol) at a dose between 2 and 6 mg/kg, followed by an intravenous administration of sodium pentobarbital at an approximate amount of 100 mg/kg.

The samples were collected from the same anatomical location of the heart for each dog, snap-frozen in liquid nitrogen, and stored at −80 °C until the date of process. Briefly, less than 25 mg of septal tissue was cut into smaller pieces on ice. Homogenization was performed in CK14-tubes (Bertin Technologies, Montigny-le-Bretonneux, France) for 2 × 20 s, with a 10-s delay in between, at 6000 rpm with the Precellys Evolution tissue homogenizer (Bertin Technologies) under cool conditions. The tissue was homogenized in lysis buffer according to the manufacturer’s recommendations for the AllPrep DNA/RNA Mini Kit (Qiagen), followed by DNA and RNA extraction according to the protocol. Samples were stored at −80 °C until further processed.

As a replication dataset, we also included myocardial tissue previously described by Cheng et al. [66] collected at several facilities in Ontario, Canada. Written consent was obtained from the clients for all procedures. Briefly, to euthanize the dogs, an intravenous injection of pentobarbital sodium at about 1 mL per pound of body weight, administered through a peripheral IV catheter. Left ventricular free wall tissue was collected from three affected Dobermanns and three unaffected large mixed-breed dogs. DCM diagnosis was made based on echocardiography (fractional shortening: unaffected 24.0 ± 7.2%, affected 7.7 ± 4.7%), gross pathology and histopathology, and CHF on the basis of history, physical examination, and thoracic radiographs. Samples were snap-frozen in liquid nitrogen and stored at −80 °C until further processed. Total RNA was prepared using TRIzol (Invitrogen, Waltham, MA, USA) as previously described [67].

### RNA libraries and sequencing

We processed the RNA samples collected in Sweden as follows: TapeStation 2200 (Agilent Technologies, Santa Clara, CA, USA) was used for quantification and integrity evaluation of the RNA samples prior to library preparation. Samples with a RIN value of 7.0 or higher were chosen for processing. Sequencing libraries were prepared from 0.5 μg of total RNA using the TruSeq® Stranded mRNA LT-sample prep kit, including poly(A) selection (Illumina Inc.). Quantification of libraries was performed with KAPA Biosystems Illumina Library Quantification Kit for ABI Prism® (Roche, Basel, Switzerland) on StepOnePlus™ Real-Time PCR Systems (Applied Biosystems, Foster City, CA, USA). Libraries were normalized to 10 nM and pooled prior to sequencing. RNA sequencing was performed using the Illumina NextSeq550 system (Illumina Inc.). Paired-end 75-bp reads were obtained, generating 1.87–2.31 Gb of data per sample.

Second, we processed the RNA samples collected in Canada as follows: 1–3 ug of total RNA was used for the isolation of poly-A RNA (Dynabeads™ mRNA purification kit, Ambion, Life Technologies, AS, Norway). The poly-A RNA was reverse transcribed to double-stranded cDNA (SuperScript™ Double-Stranded cDNA Synthesis Kit, Life Technologies, Carlsbad, CA, USA). Random hexamers (New England BioLabs, Ipswich, MA, USA) were used for priming the first strand synthesis reaction and SPRI beads (Agencourt AMPure XP, Beckman Coulter, Brea, CA, USA) for purification of cDNA. RNA-seq libraries were prepared using 60 ng of cDNA for fragmentation and tagging with Nextera™ Technology (Illumina, Inc.). After the tagmentation reaction, the fragmented cDNA was purified with SPRI beads. To add Illumina-specific bridge PCR compatible sites and enrich the library, limited-cycle PCR (5 cycles) was done according to instructions of the Nextera system with minor modifications. To generate barcoded libraries, 50 X Nextera Adaptor 2 was replaced with barcoded Illumina-compatible adapters from the Nextera Bar Codes kit (Illumina, Inc.) in the PCR setup. SPRI beads were used to purify the PCR products, and library QC was performed with Agilent Bioanalyzer (Agilent Technologies). Finally, each transcriptome was loaded to occupy 1/3 of the lane capacity in a flow cell. C-Bot (TruSeq PE Cluster Kit v3, Illumina Inc.) was used for cluster generation and Illumina HiSeq2000 platform (TruSeq SBS Kit v3 reagent kit) for paired-end sequencing with 93-bp read length.

### RNA sequencing data analysis

The generated RNA sequencing data in the FASTQ format were first checked with FastQC (version 0.11.8) [68], followed by trimming of adapter sequences and low-quality reads with Trimmomatic (version 0.32) [69]. SortMeRNA (version 2.1b) [70] was used to reduce noise from contaminating rRNAs before mapping the reads to the dog reference genome CanFam3.1 with STAR (version 2.7.2b) [71]. Of the filtered reads, 95.0–96.5% (Uppsala RNA cohort) or 86.7–88.8% (Ontario cohort) were mapped to the reference genome. We counted uniquely mapped reads with the software program featureCounts [72] and analyzed them for differential expression patterns with a generalized linear model approach and quasi-likelihood *F*-test in edgeR (version 3.10) [73] and Wald test in DESeq2 [74] with R (version 3.6.1) [55]. Finally, we inspected selected transcripts visually with Integrative Genomic Viewer (IGV, version 2.5.0) [75] and compared them with cardiac transcripts in Broad Improved Canine Annotation v1 [76] and dog mRNAs and ESTs from GenBank [77, 78].

### Transcript analyses

To verify the gene expression data obtained by RNA sequencing, we performed direct Sanger sequencing and Droplet Digital PCR (ddPCR) (Bio-Rad Laboratories, Hercules, CA, USA). Briefly, total RNA from the previous extraction of four unaffected and five affected Dobermanns from the Uppsala cohort were re-quantified using Qubit RNA BR Assay Kit (Invitrogen). A volume of 8 µl corresponding to 500 ng total RNA was used as input for cDNA synthesis with the RT2 First Strand Kit (Qiagen), according to the manufacturer’s instructions. Quantification of synthesized cDNA was performed with Qubit ssDNA Assay Kit (Invitrogen).

We used the BigDye® Direct Cycle Sequencing Kit (Applied Bioscience, Waltham, MA, USA) for direct sequencing of *RNF207*, *PRKAA2*, and *PLPP3* in three dogs to verify alternate transcripts observed in the RNA-seq data. The initial PCR amplification was performed on 4 ng cDNA using gene-specific primers (Additional file 2: Table S4) designed with Primer3 [64] tagged with M13 sequences according to the manufacturer’s instructions on the 5′-end. The following adjustment was made to the PCR protocol: 10 min enzyme activation at 95 °C, 35 cycles of denaturation for 3 s at 96 ºC, annealing at the temperature specified in Additional file 2: Table S4 for 15 s, and extension at 68 °C for 30 s. A final extension for 2 min at 72 °C was run before holding at 4 °C until further processed. Cycle sequencing was performed according to the manufacturer’s recommendations, and purification of products was done with the BigDye XTerminator® Purification Kit. Finally, we ran the samples on a 3500 Genetic Analyzer (Applied Bioscience) and evaluated the generated sequences with CodonCode Aligner v8.0.2 (http://www.codoncode.com).

We next evaluated the differential gene expression for selected candidate genes *RNF207* and *PRKAA2* and reference genes *B2M* and *SRP14* with EvaGreen-based ddPCR. Particular emphasis was placed on optimizing the ddPCR to differentiate between fragments of different lengths for exon 7 of *PRKAA2*. Briefly, QX200™ ddPCR™ EvaGreen Supermix (Bio-Rad Laboratories) was mixed with forward and reverse primers (Additional file 2: Table S4), and 5 ng cDNA/reaction. QX200™ Droplet Generation Oil (Bio-Rad Laboratories) was used to generate droplets for each sample in an Automated Droplet Generator (Bio-Rad Laboratories). The droplets were transferred to a new plate, sealed with foil, and run in a ProFlex™ (Applied Bioscience). The protocol for the PCR was initiated with an enzyme activation at 95 °C for 5 min. Forty cycles were carried out with a first step of 30-s denaturation at 95 °C, followed by annealing and extension for 1 min at primer-specific temperatures (Additional file 2: Table S4). The signal was stabilized by 5 min at 4 °C and 5 min at 95 °C, followed by infinite hold at 4 °C. Each step was ramped 2 °C/s, and the lid temperature was set to 105 °C. After the run, the plate was placed in a QX200 Droplet Reader (Bio-Rad Laboratories) for sample quantification. Data evaluation was performed in the QX Manager Software (Bio-Rad Laboratories). We normalized the expression levels to the geometrical mean of *B2M* and *SRP14* and performed statistical evaluation in JMP Pro (version 16, SAS Institute, Cary, NC, USA). Accounting for additional factors was not possible due to insufficient sample size.

### CAGE-seq data

We inspected our WGS variants in the GWAS loci in the context of the CAGE-seq data from the DoGA consortium (https://www.doggenomeannotation.org/, manuscript in preparation). This data set contains CAGE-seq data from 118 samples, spanning 36 different tissue types from 8 dogs. To review variants residing in putative enhancer sites, myocardial-expressed bidirectional CAGE-seq reads, known to indicate active enhancers [79], that resided within 1 kb of gene start sites were overlapped with the WGS variants and catalogued in the variant tables.

### Cardiac tissue stainings

Immunofluorescent staining was performed on canine cardiac tissue to inspect differences in protein expression and localization. Frozen tissue sections were cut at 4 µm, treated with acetone, and blocked with 3% BSA in PBS. ACTN2 (A7811, Sigma-Aldrich, St. Louis, MA, USA) combined with RNF207 (orb185936, Biorbyt) primary antibodies were incubated for 1 h at RT (1:250 in 1% BSA in PBS). Sections were washed with 0.5% Tween20 in PBS and incubated for 1 h at RT with Hoechst 33342 and the secondary antibodies (Invitrogen) Alexa Fluor 488 and Alexa Fluor 568. Fluorescence staining was mounted with Mowiol. Images were acquired with the Leica SP8 confocal microscope at × 63 magnification.

### Human variant analysis

To ascertain the role of *RNF207* and *PRKAA2* as potential DCM risk genes in humans, we studied three different cohorts of patients referred for diagnostic genetic testing related to a diagnosis of cardiomyopathy between 2014 and 2020 at the University Medical Center Utrecht (UMCU), the Netherlands, and at Amsterdam Medical Center (AMC), the Netherlands. Genetic testing was performed using whole-exome sequence analysis in three patient groups comprising altogether 721 people: first, a cohort of 63 patients with DCM and ventricular arrhythmias without pathogenic or likely pathogenic variants in established cardiomyopathy genes; second, a cohort of 13 patients diagnosed with arrhythmogenic cardiomyopathy without pathogenic or likely pathogenic variants in established genes; and third, 645 patients referred for diagnostic cardiovascular genetic testing. In these patients, all coding fragments and exon-intron boundaries of *RNF207* (NM_207396.3) and *PRKAA2* (NM_006252.4) were analyzed, including evaluation of pathogenicity using variant counts and MAF as presented in gnomAD version 3.1.2 [80] and prediction of impact with PolyPhen-2 [81] and SIFT [63]. Clinical information of associated patients was retrieved from medical records. The study was conducted in accordance with the principles laid out in the Declaration of Helsinki and in line with the guidelines provided by the ethics committee of the University Medical Centre Utrecht, the Netherlands.

In addition to our patient cohorts, we utilized two large-scale datasets: the UK Biobank [82] and the public data of FinnGen (freeze 8) [83]. The UK Biobank dataset was comprehensively analyzed due to the availability of individual phenotypic data, while the FinnGen data was inspected on a less detailed level. For UK Biobank data, participants were included as DCM cases if they were ever diagnosed with left ventricular systolic dysfunction (LVSD) and had not been diagnosed with coronary artery disease (CAD), valvular, or congenital heart disease more than 100 days before the report of LVSD. The definitions of LVSD, CAD, and valvular and congenital heart diseases are listed in Additional file 2: Table S5. Furthermore, we used a previously developed and validated deep-learning methodology (AI-CMR^QC^) to extract left ventricular ejection fraction (LVEF) and left ventricular end-diastolic volume (LVEDV) measurements from the cardiac magnetic resonance data [84]. Any negative values and outliers (defined as larger or smaller than the median ± 3 × interquartile range) were removed as quality control. Then, participants were also included as DCM cases if they had an LVEF below 45% and an indexed LVEDV larger than 2 standard deviations from normal [85, 86]. Variant analysis in *RNF207* and *PRKAA2* was performed as described above, and an additional analysis of variant enrichment was performed for *RNF207* by comparing the number of variants in affected and non-affected participants with Fisher’s exact test. Finally, we investigated the FinnGen data for *RNF207* and *PRKAA2* by querying each gene for phenotype-associated variants in the online interface, using a significance threshold of *p* < 5 × 10^−8^, and filtering the results for cardiovascular phenotypes.

## Results

### Two independent loci on canine chromosome 5 are associated with left ventricular systolic dysfunction and dilatation

Altogether, 540 DCM-affected and healthy Dobermanns were recruited for genetic studies. The dogs were split into three cohorts for discovery, replication, and validation (Table 2). The main cohort with 431 dogs was used for the discovery phase. The 74 samples from the Utrecht cohort served as a GWAS replication cohort. The Uppsala cohort of 35 samples was used to validate the associated variants.

We comprehensively evaluated 431 Dobermanns from Europe (main cohort) and categorized them according to echocardiographic and 24-h ECG (Holter) findings: dogs with sole echocardiographic abnormalities and no previous experience of congestive heart failure (“echo only,” *N* = 45), echocardiographic abnormalities and VPCs and no previous experience of congestive heart failure (“echo + arrhythmia,” *N* = 113), sole VPCs and no previous experience of congestive heart failure (“arrhythmia only,” *N* = 70), congestive heart failure (“CHF,” *N* = 55) and clinically healthy, mostly over 6-year-old dogs (“healthy,” *N* = 148) (Table 3). We then utilized these subcohorts to conduct univariate linear mixed model GWAS to inspect the common and separate genetic risk factors of both left ventricular systolic dysfunction and dilatation as well as VPCs. Dogs in the “healthy” subcohort were included in the GWAS cohort if they were at least 6 years old at the time of the clinical examination (*N* = 148).

**Table 3:** Summary of clinical examination results in each Dobermann subcohort of the main cohort. *N* in the “Subcohort” column refers to the number of dogs in each group, and *N* in the other columns refers to the number of dogs from whom the corresponding parameter was available

Subcohort	Metrics	Age (years)	Weight (kg)	LVEDV/BSA (ml)	LVESV/BSA (ml)	LVIDd (cm)	LVIDs (cm)	LA/Ao	FS (%)	VPC (*N*)
Healthy (*N* = 148)	*N*	148	148	148	148	148	148	148	148	148
	Mdn	8.40	35.05	72.30	36.30	4.01	2.96	1.28	26.00	4.00
	SD	1.63	5.07	10.64	7.70	11.33	2.02	0.87	5.68	29.98
	Q1	7.20	31.00	66.91	30.01	3.65	2.69	1.16	23.18	1.00
	Q3	9.40	38.40	80.45	42.62	4.23	3.24	1.35	29.50	9.00
Arrhythmia only (*N* = 70)	*N*	70	68	67	67	66	56	68	65	69
	Mdn	8.25	34.8	81.51	41.70	4.38	3.29	1.24	24.00	1602.00
	SD	2.55	5.47	12.25	9.19	15.40	0.42	0.15	6.06	7478.22
	Q1	6.00	32.20	73.13	36.49	4.12	3.00	1.17	20.00	497.00
	Q3	9.00	39.00	90.19	48.90	4.62	3.58	1.41	27.00	3966.00
Echo only (*N* = 45)	*N*	45	45	45	45	44	40	45	44	45
	Mdn	6.00	34.40	114.98	71.12	5.11	4.19	1.33	17.36	6.00
	SD	2.94	7.00	25.79	22.27	15.18	0.59	0.29	5.12	29.03
	Q1	4.00	32.40	102.75	61.74	4.83	3.80	1.21	13.48	3.00
	Q3	8.00	42.90	130.85	89.60	5.53	4.60	1.47	21.00	21.00
Echo + arrhythmia (*N* = 113)	*N*	113	106	101	100	100	98	101	100	110
	Mdn	8.00	38.00	109.50	69.00	5.04	4.12	1.35	17.31	563.00
	SD	2.50	5.31	27.42	25.13	7.30	0.79	0.27	6.79	6117.55
	Q1	6.00	34.20	100.51	60.71	4.74	3.79	1.27	12.75	165.00
	Q3	9.00	41.20	132.61	84.89	5.53	4.64	1.44	21.00	3475.00
CHF (*N* = 55)	*N*	55	52	54	54	54	54	53	54	52
	Mdn	7.00	34.95	159.16	116.65	6.09	5.12	2.07	13.55	835.00
	SD	2.33	5.62	46.55	42.22	0.75	0.82	0.61	6.50	41,535.74
	Q1	5.256	31.90	133.80	91.26	5.49	4.53	1.82	9.24	136.00
	Q3	9.00	40.00	196.10	139.16	6.44	5.68	2.49	17.26	2781.00

*BSA* body surface area, *CHF* congestive heart failure, *FS* fractional shortening, *LA/Ao* left atrial to aortic root diameter ratio, *LVEDV* left ventricular end-diastolic volume, *LVESV* left ventricular end-systolic volume, *LVIDd* left ventricular internal diameter end-diastole, *LVIDs* left ventricular internal diameter end-systole, *Mdn* median, *Q1* lower quartile, *Q3* upper quartile, *SD* standard deviation, *VPC* ventricular premature complex

After conducting quality control, we performed various analyses with different subcohorts. Analyses focused on the typical DCM phenotype with subcohorts “echo only; echo + arrhythmia” and “echo only; echo + arrhythmia; CHF” yielded a genome-wide significant signal at chr5:60.5 Mb; however, VPC-oriented analyses with subcohorts “arrhythmia only” and “arrhythmia only; echo + arrhythmia” did not replicate the association on chromosome 5 despite the similar sample sizes (Additional file 1: Figs. S1 and S2, Additional file 2: Table S6). We, therefore, conducted an extended analysis to explore the chromosome 5 locus with an echocardiography-focused cohort, composed of cases from the “echo only,” “echo + arrhythmia,” and “CHF” subcohorts as well as an independent cohort of affected Dobermanns with less comprehensive clinical evaluation (“Utrecht cohort”). This analysis further strengthened the association (Fig. 1), which consists of two loci, one at 60 Mb (“major locus”) and the other at 53 Mb (“minor locus”). As indicated by the lack of SNP correlation between the loci, the signals are independent. Two orders of magnitude more significant than the minor locus, the major locus spans a 2.3 Mb region at 59.2–61.5 Mb with 46 *M*_eff_-significant SNPs and a relatively long homozygous haplotype around the top SNP (chr5:60,531,090). In contrast, the structure of the region around the minor locus, whose top SNP is at chr5:53,109,178, is less apparent (Fig. 1d, e). Our locus partially overlaps the previous findings of Mausberg et al. [35] who discovered a region associated with left ventricular systolic dysfunction and dilatation as well as VPCs in Dobermanns, with the most significant signal at chr5:50–51 Mb (canFam2 chr5:53–54 Mb) [35].

To assess the mechanisms and potential interaction of the risk SNPs at chr5:53,109,178 and chr5:60,531,090, we performed logistic regression in R using the “echo only,” “echo + arrhythmia,” and “CHF” as cases and the “healthy” subcohort as controls. Genotypes at both SNPs and sex were significantly associated with the echocardiographic phenotype (Additional file 2: Tables S7 and S8). First, male dogs were more likely affected by left ventricular systolic dysfunction and dilatation than female dogs (OR = 2.88, *P* < 0.001, df = 1). Second, genotype at chr5:53,109,178 increased the likelihood of echocardiographic changes: dogs with G/G were more likely affected than dogs with G/A (OR = 3.29, *P* < 0.01, df = 1) and A/A (OR = 9.07, *P* < 0.001, df = 1) genotypes, and dogs with G/A were more likely affected than dogs with A/A (OR = 2.75, *P* < 0.001, df = 1; Fig. 1h). In contrast, genotype at chr5:60,531,090 seemed to act in a recessive manner: A/A homozygotes were more likely affected than G/A heterozygotes (OR = 6.78, *P* < 0.001, df = 1) and G/G wild types (OR = 10.93, *P* < 0.001, df = 1; Fig. 1i). There was no significant difference between heterozygous and wild-type dogs (OR = 1.61, *P* = 0.064, df = 1). Finally, the interaction between chr5:60,531,090 and chr5:53,109,178 could not be tested due to a partial separation of data points; however, a frequency plot of case status by joint genotypes did not indicate the presence of an interaction but rather an additive effect (Fig. 1j).

Lastly, we assessed the role of previously indicated Dobermann and human DCM loci and genes in our GWAS cohort. Other than the redefinition of the chr5:50–51 Mb locus described above [35], we did not detect any signal at the previously claimed Dobermann DCM loci at chr14:20.9 Mb (*PDK4*, known as “DCM1” gene test) [39] and chr36:22.3 Mb (*TTN*, known as “DCM2” gene test) [38] or regions corresponding to human DCM loci at canine chr20:29.8 Mb (human *BAG3,* rs2234962) [87], chr2:81.6 Mb (human *HSPB7*, rs10927886) [88], chr20:4.3 Mb (human chr3p25.1, rs62232870) [6], and chr26:28.6–28.8 (human chr22q11.23) (Additional file 1: Fig. S3).

Our results reveal two independent loci, one novel and one redefined, associated with left ventricular systolic dysfunction and dilatation in Dobermanns. The risk loci act with different mechanisms, emphasizing the complex genetic background of DCM in Dobermanns and revealing major risk alleles with potential use as a joint marker test in dogs. We further show that VPCs are not associated with the loci on chromosome 5, highlighting a distinct genetic etiology for the typical DCM phenotype.

### Prioritization highlights three candidate genes in the chromosome 5 loci

We investigated the gene content of the major and minor loci and their flanking regions. The total number of genes was 80 in the major locus region (chr5:58.2–63.3 Mb; resembling GRCh38_1:3.6–10.2 MB) and 32 in the minor locus region (chr5:50.0–54.0 Mb; resembling GRCh38_1:56.1–60.0 MB). To determine the candidate genes in each locus, we systematically evaluated the genes according to their type (protein-coding or other), expression in ventricular septal heart tissue of five Dobermanns affected by DCM and four unaffected Dobermanns (“Uppsala RNA cohort”), expression of an orthologous human gene in heart muscle in Human Protein Atlas (HPA) [89] and left ventricle in Genotype-Tissue Expression project (GTEx) [90], and the number of filtered genomic variants in ten whole-genome sequenced affected Dobermanns (five from the “echo only” and five from the “echo + arrhythmia” subcohorts) (Additional file 2: Tables S9 till S13). In the first step, we retained only those genes that (1) were protein-coding, (2) were expressed in ventricular septal heart tissue in the unaffected Dobermanns, (3) had a human orthologue expressed in the target tissues in HPA and GTEx, and (4) had at least one variant of any type in the Dobermann WGS data. This procedure yielded 22 genes, of which eight resided in the minor locus: *HOOK1*, *FGGY*, *MYSM1*, *OMA1*, *DAB1*, *FYB2*, *PRKAA2,* and *PLPP3*; and 14 in the major locus: *NPHP4*, *KCNAB2*, *CHD5*, *RNF207*, *GPR153*, *ACOT7*, *HES2*, *NOL9*, *DNAJC11*, *CAMTA1*, *VAMP3*, *ERRFI1*, and *RERE*.

We next analyzed these 22 preliminary genes for potentially pathogenic variants or expression changes based on RNA-seq data from the Uppsala RNA cohort (*N* = 9). Differential expression analysis did not reveal significant quantitative differences in these genes between affected and unaffected Dobermanns, so we utilized the data to inspect the genes for qualitative changes. Therefore, we included genes that (1) had variants in exons or at splice sites or (2) indicated aberrant splicing when visualized with Integrative Genomics Viewer (IGV) [42]. First, exonic or splice site variants were present in five genes: *MYSM1*, *FYB2*, *PLPP3*, *RNF207*, and *GPR153*. As the exonic variants in *MYSM1* and *PLPP3* were predicted to be synonymous and the nonsynonymous variants in *FYB2* and *GPR153* to be tolerated with SIFT scores of 0.45 and 0.2, only *RNF207* was retained based on genomic variants. Second, aberrant splicing was detected in *RNF207*, *PRKAA2*, and *PLPP3*. Thus, our final list included three candidate genes: *RNF207*, *PRKAA2*, and *PLPP3*.

### RNA analyses confirm aberrant splicing of *RNF207* and *PRKAA2*

**Fig. 2:**
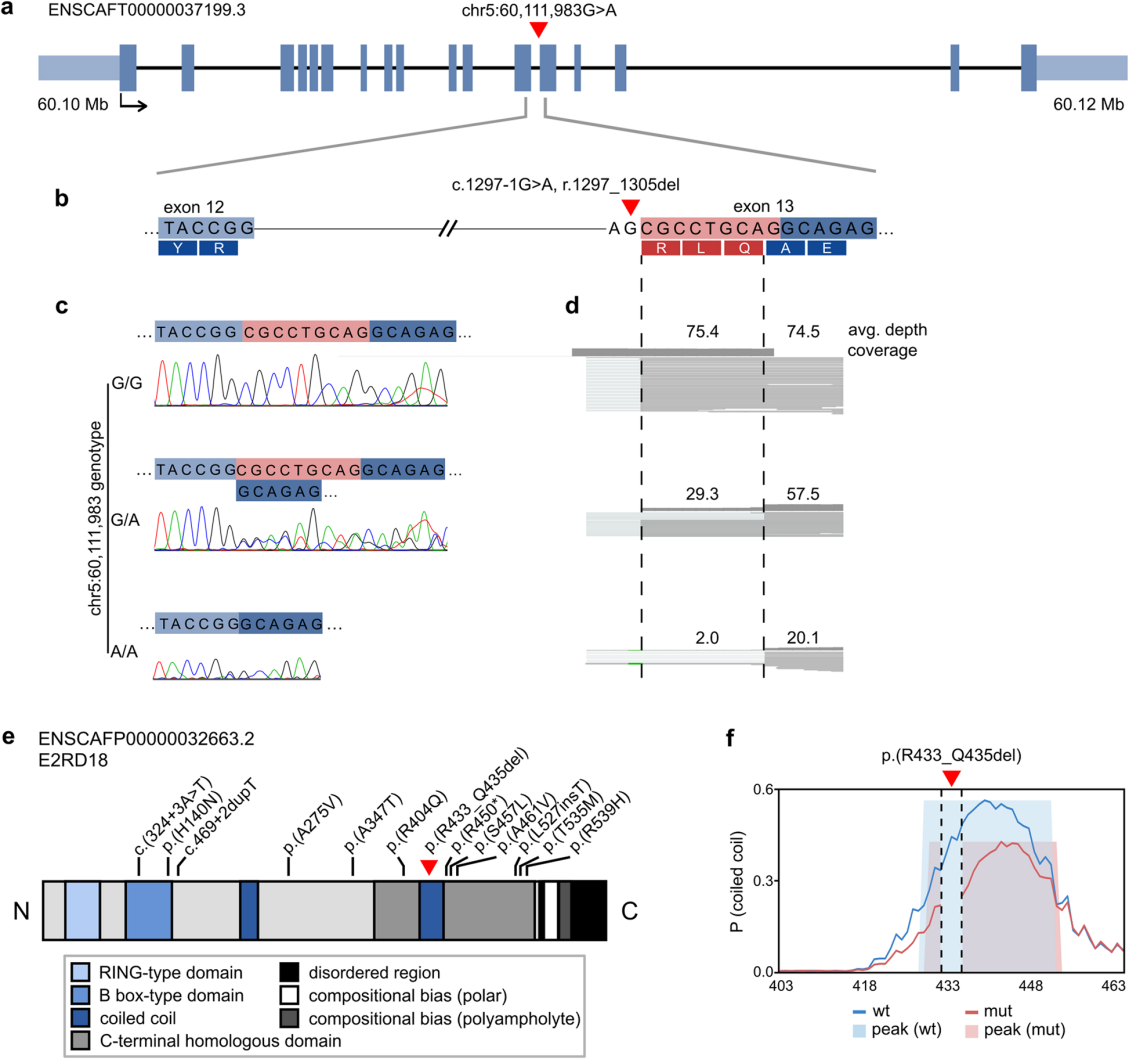
Alternative splicing and potentially damaging human variants in *RNF207*. The position of the canine *RNF207* splice variant (chr5:60,111,983G > A) is indicated with a red triangle. **a** Exon-intron structure of the canine *RNF207* transcript (ENSCAFT00000037199.3). **b** The location of the variant at the splice acceptor site of exon 13 (ENSCAFT00000037199.3:c.1297-1G > A, r.1297_1305del). **c** Representative chromatograms of *RNF207* cDNA in Dobermanns with different chr5:60,111,983 genotypes. The first nine bases of exon 13 are skipped in dogs with at least one copy of the A allele. **d** The average read depth and coverage at the variant site in the mRNA-seq data of the same Dobermanns as in **c**. Reads without the first nine bases of exon 13, indicated within the dashed lines, are observed in dogs with at least one copy of the A allele. Two reads that likely comprised pre-mRNA are observed at the deletion site in the A/A dog. **e** A schematic representation of the canine RNF207 protein (ENSCAFP00000032663.2, E2RD18). The p.(R433_Q435del) variant is indicated with a red triangle, and positions corresponding to protein-changing, potentially damaging variants in human cardiomyopathy patients with black lines. **f** Coiled-coil conformation of the wild type and p.(R433_Q435del) sequences at residues 403–463 as predicted by DeepCoil2. The coiled-coil domain may be affected by the loss of residues 433–435 in the altered protein. The propensity of coiled-coil conformation and detected peaks are indicated in blue and light blue for the wild-type sequence and in red and pink for the mutated sequence

We further explored our three candidate genes, *RNF207*, *PRKAA2*, and *PLPP3*, for aberrant splicing in the Uppsala RNA cohort. First, the mRNA sequencing data revealed a loss of the first nine bases of exon 13 (r.1297_1305del) in samples with the *RNF207* splice site variant (chr5:60,111,983G > A, c.1297-1G > A) (Fig. 2). The genotype of the variant correlated with splicing: wild types (G/G) showed solely normal transcripts, while heterozygotes (G/A) expressed both the normal and the altered *RNF207* transcripts, and the only homozygote mutant (A/A) showed no wild-type transcripts. In the RNA-seq data of the homozygous dog, two reads likely comprised pre-mRNA [43] included part of intron 12; this was not observed in the *RNF207* cDNA Sanger sequence. Quantitative changes in *RNF207* mRNA expression between cases and controls were inspected in the RNA-seq data and with Droplet Digital PCR (ddPCR), but no differences were observed.

We next studied the reading frame of the predicted RNF207 mutant protein with ORFfinder [91], which did not indicate a shift of the open reading frame. However, the r.1297_1305del variant in exon 13 is predicted to delete three amino acid residues R, L, and Q (p.(R433_Q435del)). The L and Q residues are conserved in 94 Eutherian sequences (Additional file 2: Table S14), and the deletion is located within a coiled-coil domain (Fig. 2e), the conformation of which may be affected by p.(R433_Q435del) and subsequent interruption of the heptad repeat as indicated by DeepCoil2 [92].

As the splicing-linked chr5:60,111,983G > A variant was present in both the sequencing data and on the GWAS SNP array, we conducted a combined evaluation of large canine populations to explore the association of the variant with the disease across cohorts and to investigate its breed distribution. A Dobermann cohort of 35 dogs (“Uppsala cohort”), containing the 9 Uppsala RNA samples and including a total of 24 dogs affected by left ventricular systolic dysfunction and dilatation and 11 unaffected dogs, supported the association with a *P*-value of 0.002 (*χ*^2^ = 9.43, df = 1). Consequently, the association of this variant with echocardiographic changes in the entire echocardiographic Dobermann cohort (“echo only,” “echo + arrhythmia,” “CHF,” and “healthy” subcohorts; Utrecht cases; and the Uppsala cohort) was highly significant (*P* = 1.72 × 10^−11^, *χ*^2^ = 45.26, df = 1). In the GWAS, the variant was the fourth most significant SNP, with a negligible difference from the top SNP (*p*_raw_ = 3.34 × 10^–9^). Next, we inspected the presence of the variant in the WGS dataset utilized in variant filtering and in an independent cohort of 14,230 dogs genotyped on a commercial platform (Wisdom Panel™, MyDogDNA™, and Optimal Selection™ canine genetic testing products by Wisdom Panel (Portland, OR, USA)). Of the 403 whole genomes, excluding three samples that were not called, 86.3% were wild-type, 9.8% heterozygous, and 3.9% homozygous. In addition to Dobermanns, heterozygotes were found in 15/88 and homozygotes in 4/88 breeds (Additional file 2: Table S15). Similar frequencies were observed in the commercial cohort: 87.7% were wild type, 9.4% heterozygous, and 2.9% homozygous, with heterozygotes in 113/320 and homozygotes in 55/320 breeds or breed varieties (Additional file 2: Table S16). Together, these data show that the chr5:60,111,983G > A variant is strongly associated with the typical DCM phenotype in Dobermanns, occurs across breeds, and is not of recent origin.

**Fig. 3:**
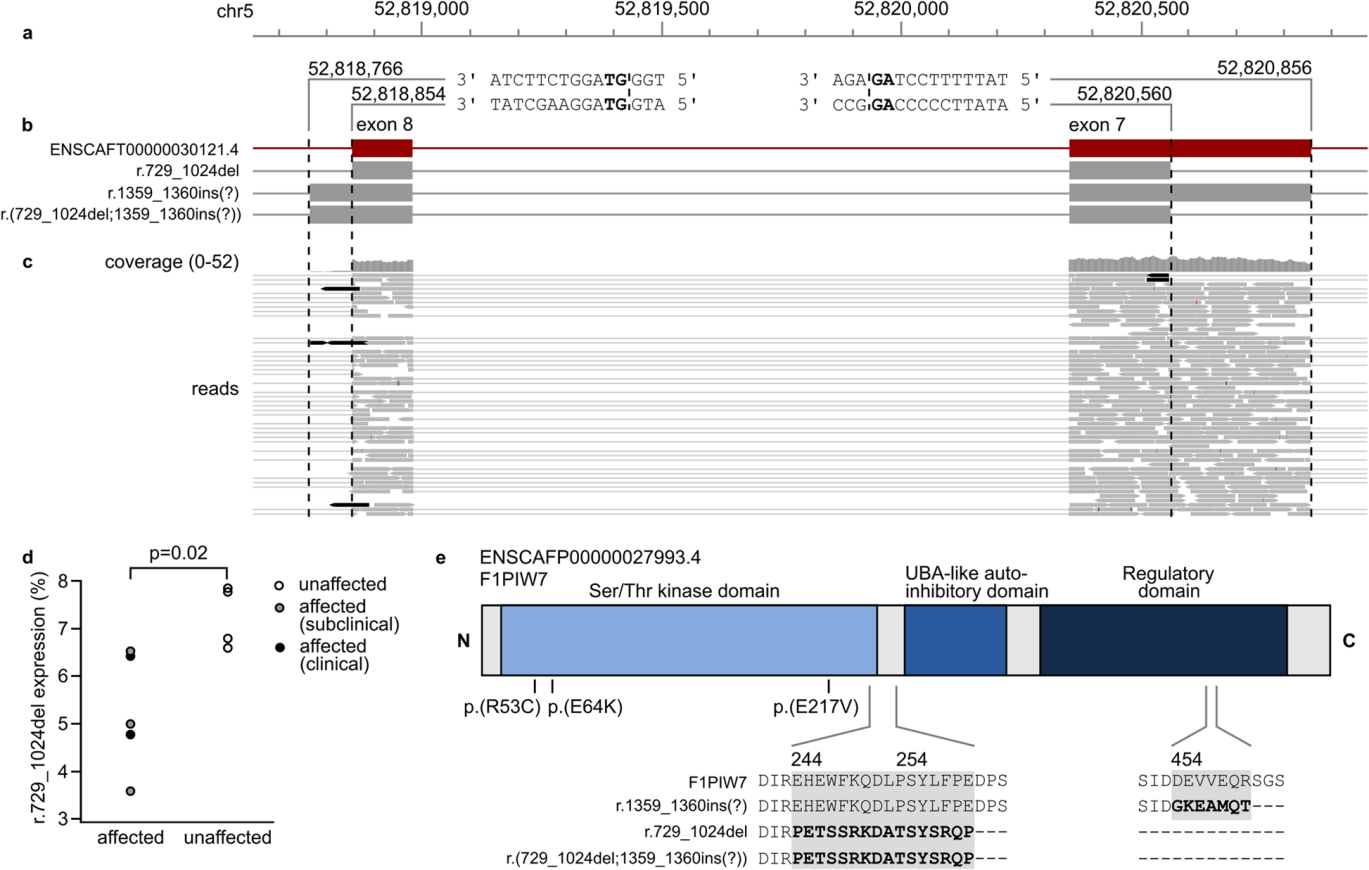
Alternative splicing and potentially damaging human variants in *PRKAA2*. **a** Sequences of splice sites observed in canine RNA-seq data. The canonical GT and AG splice site sequences at the exon-intron junctions are indicated in bold. **b** Schematic representation of canine *PRKAA2* transcripts and boundaries of exons 7 and 8: ENSCAFT00000030121.4, exon 7 deletion (chr5:52,820,560–52,820,856, r.729_1024del) mutant, intron 8 retention (chr5:52,818,766–52,818,853, c.1359-1360ins(?)) mutant, and a predicted transcript with both aberrant splice sites (r.(729_1024del;1359_1360ins(?))). **c** Alternate exon 7 and 8 splicing in the mRNA-seq reads of one affected Dobermann. The aberrant reads are indicated in black. **d** Proportional expression of the exon 7 mutant transcript detected with ddPCR in five DCM-affected and four unaffected Dobermanns. **e** Schematic representation of the canine PRKAA2 protein (ENSCAFP00000027993.4, F1PIW7) and the predicted amino acid sequences of the aberrant transcripts. The r.729_1024del variant is predicted to result in truncation upstream of the UBA-like autoinhibitory domain, and the c.1359-1360ins(?) transcript is predicted to contain a premature stop codon before the C-terminal regulatory domain involved in heterotrimerization. Positions corresponding to potentially damaging variants in human cardiomyopathy patients are indicated with black lines. ENSCAFT00000030121.4 was used as a reference sequence for the mutant transcripts, and domain annotation was obtained from CDD

We next inspected the mRNA sequencing data of *PRKAA2* and identified reads indicating aberrant splicing of exons 7 and 8 (Fig. 3). First, we observed a deletion in exon 7 (chr5:52,820,560–52,820,856, r.729_1024del) in three affected and one healthy dog. Second, we detected a partial retention of at least 87 bp at the start of intron 8 (chr5:52,818,766–52,818,853, r.1359-1360ins) in all samples. These splice events were also observed in an independent cohort of three DCM-affected Dobermanns and three unaffected large mixed-breed dogs (“Ontario cohort”). Notably, both the reference and aberrant splice sites match the canonical GT-AG splice site sequence. Evaluation with ORFfinder predicted a premature stop codon for both aberrant splicing events, resulting in truncation and loss of highly conserved domains. We confirmed the expression of the r.729_1024del transcript in all dogs of the Uppsala RNA cohort with ddPCR, which showed proportionally lower expression (ANOVA, *P* *= *0.02, df = 8) of the aberrant transcript in DCM-affected dogs (Fig. 3d).

To explore how the variant architecture of *PRKAA2* could be linked to the aberrant transcripts, we whole-genome sequenced one unaffected and two affected Dobermanns from the Uppsala RNA cohort. We performed a targeted analysis on exons 7–8 and introns 6–8 (chr5: 52,817,157–52,828,472) to discover variants present in the two affected dogs, absent in the unaffected dog, and present in less than 10% of the control genomes. No variants in the region passed the analysis criteria.

Finally, we assessed the splicing of *PLPP3* with cDNA Sanger sequencing. The aberrant splicing detected with IGV visualization was not present in the cDNA; instead, the sequence matched completely to *PLPP3* transcript 201 (ENSCAFT00000065003.1). Due to a lack of quantitative or qualitative transcriptomic changes, we discarded *PLPP3* from further follow-up.

In summary, our RNA analyses highlight alternatively spliced transcripts in *RNF207* and *PRKAA2* and suggest that aberrant RNA processing may be involved in the molecular mechanisms of DCM in Dobermanns. While the *RNF207* splice site variant was demonstrated to result in an altered transcript, no clear candidate variants explaining the aberrant splicing of *PRKAA2* were found.

### Characterization of RNF207 protein in myocardial samples

**Fig. 4:**
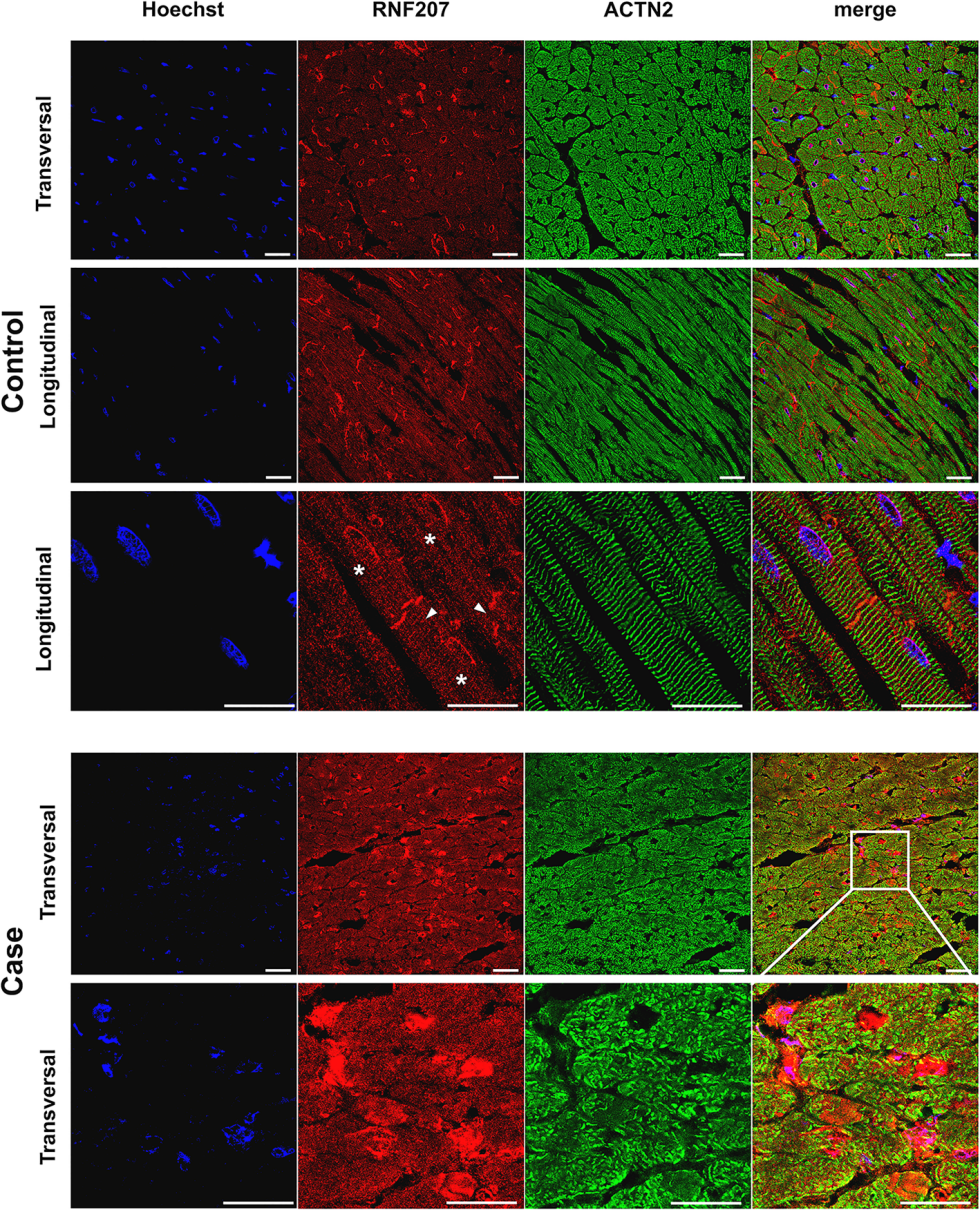
Immunofluorescent staining of RNF207 in canine cardiac tissue. Healthy canine non-Dobermann cardiac tissue (top three rows) was stained for RNF207 to detect the differences in the expression or localization associated with disease and genotype. Additionally, tissue was stained for Hoechst in blue to show the nuclei and for actinin alpha 2 (ACTN2) in green to visualize the sarcomere structures. Localization is indicated from a transversal and longitudinal perspective. RNF207 is expressed in the cytoplasm of cardiomyocytes, intercalated disks (arrows), and perinuclearly (asterisks). In the cardiac tissue of the single DCM case homozygous for the RNF207c.1297-1G > A variant, a patchy expression resembling cellular mosaicism was detected (bottom row)

To further examine the role of RNF207, canine cardiac tissue was used for immunofluorescent staining of the RNF207 protein. In the control tissue of non-Dobermann dogs, a positive signal was observed in the cytoplasm with a perinuclear distribution (asterisks) and in the intercalated disks (arrows) (Fig. 4, top three rows). The tissue obtained from Dobermann cases and controls was more damaged due to freezing artifacts, complicating the signal quantification. Only one case homozygous for the *RNF207* c.1297-1G > A variant was available (Fig. 4, bottom two rows). This single case showed patches of cellular mosaicism and differences in RNF207 protein expression, even within single cardiomyocytes, which was not or barely observed in the controls.

### Analysis of *RNF207* and *PRKAA2* in human cohorts reveals enrichment of *RNF207* variants in DCM cases

To follow-up on the canine discoveries, we inspected variants in *RNF207* and *PRKAA2* in three human cohorts: first, 721 cardiomyopathy patients that underwent diagnostic cardiovascular whole-exome-based genetic testing; second, UK Biobank participants affected by DCM; and third, the FinnGen Consortium (https://r8.finngen.fi/). To investigate the variant architecture of the genes, we evaluated the variants in coding regions and exon-intron boundaries of *RNF207* and *PRKAA2* and retained those with predicted damaging impact on the protein (Additional file 2: Tables S17 and S18). As a result, we discovered 12 variants in *RNF207* and three in *PRKAA2*. First, of the *RNF207* variants, nine were missense, one was an in-frame insertion, one a single-base duplication in a splice donor, and one an SNV at a splice site. Notably, the c.324 + 3A > T and p.(R404Q) variants were present in two patients, and p.(R450*) in three patients. Second, in *PRKAA2*, all three variants were missense. Collectively, 5/19 variants were absent in gnomAD [80], while the rest showed variable frequencies, with the highest population-specific MAFs ranging between 0.001 and 1.4% (median: 0.4%).

To test the association of the identified variants with DCM, we conducted Fisher’s exact test. Due to sampling bias in the cardiomyopathy patient cohort, we only included the participants and variants in the UK Biobank cohort. As no variants in *PRKAA2* passed our inclusion criteria in the UK Biobank cohort, we performed the analysis solely for *RNF207*. The seven *RNF207* UK Biobank variants were present in 7 DCM cases and 874 controls, and the number of DCM cases and controls without these variants were 350 and 199,735, respectively. This indicated significant enrichment of the variants in DCM-affected participants (*p* = 0.0011).

We additionally investigated *RNF207* and *PRKAA2* in the public dataset of the FinnGen consortium (freeze 8, https://r8.finngen.fi/) [83]. Using a significance threshold of 5 × 10^−8^, we queried each gene for any variants associated with cardiovascular phenotypes. *RNF207* had no significant variants, and *PRKAA2* was linked to several phenotypes involving myocardial ischemia, hypertension, and lipid metabolism. While these results did not indicate additional DCM-associated variants, they support the involvement of *PRKAA2* in cardiac functions.

Based on the Netherlands and UK Biobank cohorts, our findings suggest the involvement of *RNF207* in human DCM. Due to the relatively high population-specific MAFs, the variants are unlikely to have large effect sizes.

## Discussion

Dilated cardiomyopathy is often a fatal heart disorder with no cure, and a substantial part of patients have a genetic etiology. While general treatment options exist, personalized medicine could improve the outcome [93]; however, differentiating between the primary molecular drivers and secondary compensatory responses is challenging, and genetic information is essential in thoroughly dissecting the pathophysiological mechanisms. Our study utilized a spontaneous canine model of DCM, with clinical characteristics and sex predisposition similar to human DCM, to map novel loci and candidate genes. Our approach was facilitated by an inbred population of Dobermann dogs with extensive LD, which allowed us to overcome obstacles caused by clinical heterogeneity in human populations.

The genomic and transcriptomic data reveal a two-locus model for canine DCM, document *RNF207* and *PRKAA2* as novel candidate genes, and highlight putative pathophysiological mechanisms via perturbed mRNA expression caused by aberrant splicing. We further demonstrate that a distinct genetic etiology underlies the typical DCM phenotype but not VPCs as a sole abnormality in Dobermanns, challenging the current understanding of Dobermann DCM as a singular disorder with varying clinical signs. Our study provides a polygenic animal model with the potential for an increased understanding of ventricular morphology, myocardial metabolism, electrophysiology, and molecular pathophysiology.

The first novel candidate gene, *RNF207*, encodes a RING finger protein with a proposed function in regulating cardiac action potential duration. The gene was previously associated with extended QT interval in human GWAS studies [94–96], and overexpression in a rabbit cardiomyocyte model resulted in a shortened action potential [97]. A recent study further reported that overexpression exacerbated and knockdown attenuated cardiac hypertrophy in a mouse model with transverse aortic constriction [98]. Interestingly, RNF207 was shown to interact with TAB1, a protein that activated pathways related to cardiac hypertrophy [98] and, in another study, accumulated in nuclei and intercalated disks of isolated rat cardiomyocytes [99]. By using canine myocardium, we showed for the first time that RNF207 is expressed not only in the cytoplasm of cardiomyocytes and perinuclearly but also in intercalated disks. Therefore, the local expression in the intercalated disk is a novelty that fits the role of RNF207 in established cardiac processes. Gap junctions in the intercalated disks allow the passage of ions between cells and facilitate synchronized cardiomyocyte contraction; importantly, altered cell-cell connections and a scattered structure of intercalated disks have been linked to DCM, including more arrhythmic subforms [100–102]. Although cardiac tissue was available from only one DCM-affected dog homozygous for *RNF207* c.1297-1G > A, we observed remarkable cellular mosaicism for RNF207 in cardiomyocytes. This phenomenon, recently described as a stochastic allelic expression in DCM [103], has been reported in hypertrophic cardiomyopathy patients with variants in myosin heavy chain 7 (*MYH7*) [104–106] and myosin-binding protein C3 (*MYBPC3*) [107–109].

The second candidate gene, *PRKAA2*, is an established energy sensor in cardiac muscle. As a subunit of the heterotrimeric AMP-activated kinase (AMPK) complex, *PRKAA2* (also known as AMPKα2) regulates glucose and lipid metabolism, especially during ischemia [110–113]. In Dobermanns, we observed differential expression of an aberrant *PRKAA2* transcript in DCM-affected and unaffected dogs; however, we did not identify a genomic variant linked to the splicing event. While the GWAS findings showed a significant association near *PRKAA2*, we could not identify filtered genomic variants linked to splicing; therefore, we cannot exclude the possibility of the differential transcript usage being a secondary response to DCM itself, or simply a result of canonical GT and AG splice site motifs present at the observed splice sites. Nevertheless, our results suggest *PRKAA2* as a possible new DCM gene to be explored in future research.

Our discoveries in the canine model allowed us to perform a targeted analysis of our newly identified candidate genes in human DCM patients. We observed 15 potentially pathogenic variants in *RNF207* and *PRKAA2*, highlighting their relevance in understanding human DCM. In the UK Biobank cohort, *RNF207* variants were significantly enriched in DCM-affected participants. The reasonably high minor allele frequencies of most variants indicate they would likely have small effect sizes; this is in line with the important role of common genetic polymorphisms in human DCM [114]. After validation, our candidate genes could explain some of the missing genetic backgrounds for the disease. This project is an outstanding example of how a spontaneous canine patient population can aid in understanding the pathophysiology of DCM without needing to induce the disease, as is often the case in animal models. Studying this naturally occurring disease in dogs helps us understand essential factors in disease progression, like aging. The complex genetic cause of disease against a more homogeneous background due to inbreeding enables the discovery of minor genes involved in DCM. Comprehensive One Health approaches in the future will lead to a better understanding of DCM and have the potential to lead to better overall care and treatment [13].

Although we present a multi-omics study in a canine model of DCM with the largest cohort to date, we recognize several limitations. First, extensive clinical information was missing in part of our study population, particularly with older samples collected decades ago. We, therefore, split our population into a main cohort consisting of 431 individuals and a replication cohort with 74 less extensively phenotyped individuals. This way, we avoided the loss of statistical power and enabled the validation of the associated signals. Second, the sample size of the VPC-oriented cohort remained too small to reach a genome-widely significant signal. Third, we did not have optimal fresh tissue available for transcript analyses. Instead of the ventricle tissue, we had to use septal tissue to study splicing effects. However, our transcripts of interest were expressed also in septal tissue and did not prevent conclusions. In the future, a larger biobank of relevant cardiac tissues, including the ventricular tissue, should be collected to understand pathophysiological adaptation. This will also be instrumental in validating the cellular mosaicism in RNF207 protein expression, which was detected in our sole patient homozygous for *RNF207* c.1297-1G > A. Finally, validation of this expression in human cardiac tissue of variant carriers, which we did not have in our biobank [115], would confirm the role of RNF207 in DCM.

Our results may also impact veterinary diagnostics, as we introduce a potential two-marker gene test for Dobermann DCM. Notably, our findings confirmed and refined a previously reported DCM locus on chromosome 5 [35] but did not replicate the association of the previously proposed and widely tested DCM-linked loci at *PDK4* [39] and *TTN* [38] discovered in American Dobermanns. Whether this reflects potential differences between the American and European Dobermann populations or suggests population-independent issues in the clinical validity of the *PDK4* and *TTN* gene tests cannot be concluded; however, the loci were not predictive of disease risk in our large European cohort. The cumulative prevalence of the disorder in the breed has been reported to be almost 60% and average disease onset past the typical age at breeding [18], and eradicating the disease from the breed will therefore be exceedingly difficult without a prognostic gene test. While not completely predictive, our GWAS results illustrate the significant risk genotypes and could after thorough validation, be utilized for excluding dogs at the highest risk from the breeding pool, resulting in significantly improved animal health and welfare. Genetic evaluation could also help to stratify the affected dogs that would best benefit from current and future treatment plans.

## Conclusions

Our dual-species multi-omics approach revealed *RNF207* and *PRKAA2* as two novel genetic risk factors involved in DCM. Our study establishes an important large bidirectional animal model of DCM for potential preclinical studies and drug development. The shared clinical characteristics and molecular etiology between humans and dogs enable the translation of canine studies to human medicine, making the affected breed relevant in accelerating discoveries in cardiac research.

### Supplementary Information

**Additional file 1: Fig. S1.** Manhattan plots from GWAS analyses with different subcohort combinations of affected Dobermanns. **Fig. S2.** Multi-dimensional scaling (left) and quantile-quantile plots (right) from GWAS analyses with different subcohort combinations of affected Dobermanns. **Fig. S3.** Locus plots from an extended GWAS analysis with 235 cases from the “echo only”, “echo + arrhythmia” and “CHF” subcohorts and Utrecht cohort, and 143 controls from the “healthy” subcohort.

**Additional file 2: Table S1.** Clinical parameters, cohort classification and chr5:60,111,983 genotypes of 35 Dobermanns evaluated in Uppsala, Sweden. **Table S2.** A list of 393 public or in-house genomes from wolves and 87 dog breeds used in filtering and variant screening. **Table S3.** Distribution of the chr5:53,109,178G>A variant in 321 array genotyped Dobermanns, 10 Dobermann whole genomes and 393 non-Dobermann whole genomes. **Table S4.** Primer sequences, total concentration in reaction (Conc.), annealing temperature (An. temp) and assays used in transcript analyses. **Table S5.** Phenotype definitions used with UK Biobank data. CAD = coronary artery disease, LVSD = left ventricular systolic dysfunction. **Table S6.** Results of various univariate linear mixed model association analyses in GEMMA with different case subcohorts. **Table S7.** Association of sex and risk SNPs chr53:109,178 and chr5:60,531,090 with echocardiographic changes in the logistic regression analysis. **Table S8.** Association of sex and risk SNPs chr53:109,178 and chr5:60,531,090 with echocardiographic changes. Cases from the replication cohort were included. **Table S9.** Variants in the minor locus region (chr5:50-54 Mb) shared by two DCM-affected Dobermanns with A/A at the risk SNP chr5:53,109,178 and present in less than 10 % of 366 non-Dobermann genomes. **Table S10.** Variants in the minor locus region (chr5:50-54 Mb) shared by three DCM-affected Dobermanns with G/G at the risk SNP chr5:53,109,178 and present in less than 10 % of 366 non-Dobermann genomes. **Table S11.** Variants in the major locus region (chr5:58.2-63.3 Mb) shared by eight DCM-affected Dobermanns with A/A (*N* = 7) or G/A (*N* = 1) at the risk SNP chr5:60,531,090 and present in less than 10 % of 366 non-Dobermann genomes. **Table S12.** Gene content and annotation of the minor locus and flanking regions (chr5:50.0-54.0 Mb). **Table S13.** Gene content and annotation of the major locus and flanking regions (chr5:58.2-63.3 Mb). **Table S14.** Partial amino acid sequences and conservation of 94 Eutherian RNF207 orthologs. **Table S15.** Distribution of the chr5:60,111,983G>A variant in 9 RNA sequenced Dobermanns, 10 whole-genome sequenced Dobermanns and 393 non-Dobermann whole genomes from wolves and 87 dog breeds. **Table S16.** Distribution of the chr5:60,111,983G>A variant in 14230 dogs from 319 breeds or breed varieties genotyped on a commercial platform. **Table S17.** Potentially damaging variants in RNF207 identified in cardiomyopathy patients (UMCU and AMC), in UK Biobank participants diagnosed with DCM (UKBiobank) or in cardiomyopathy patients in the FinnGen consortium. **Table S18.** Potentially damaging variants in PRKAA2 identified in cardiomyopathy patients (UMCU and AMC), in UK Biobank participants diagnosed with DCM (UKBiobank) or in cardiomyopathy patients in the FinnGen consortium.

## Data Availability

The Dobermann WGS data has been deposited in NCBI’s SRA under a bioproject PRJNA999497. Canine RNAseq data from the University of Uppsala has been deposited in EBI Biostudies, E-MTAB-12151, and from the University of Guelph in NCBI’s PRJNA99949. Human WGS data will not be published to protect the confidentiality of the participants.
