# Supplementary material for: Identification of novel genetic risk factors of dilated cardiomyopathy: from canine to human
Source: Genome Med. 2023 Sep 18;15:73. doi: 10.1186/s13073-023-01221-3 (PMC10506233; doi:10.1186/s13073-023-01221-3)
Supplement: Supplementary file 1 — Additional file 1: Fig. S1. Manhattan plots from GWAS analyses with different subcohort combinations of affected Dobermanns. Fig. S2. Multi-dimensional scaling (left) and quantile-quantile plots (right) from GWAS analyses with different subcohort combinations of affected Dobermanns. Fig. S3. Locus plots from an extended GWAS analysis with 235 cases from the “echo only”, “echo + arrhythmia” and “CHF” subcohorts and Utrecht cohort, and 143 controls from the “healthy” subcohort. [file 13073_2023_1221_MOESM1_ESM.pdf]

Additional file 1

Identification of novel genetic risk factors of dilated cardiomyopathy: from canine to human

Julia E. Niskanen, Åsa Ohlsson, Ingrid Ljungvall, Michaela Drögemüller, Robert F. Ernst, Dennis Dooijes, Hanneke W. M. van Deutekom, J. Peter van Tintelen, Christian J. B. Snijders Blok, Marion van Vugt, Jessica van Setten, Folkert W. Asselbergs, Aleksandra Domanjko Petrič, Milla Salonen, Sruthi Hundi, Matthias Hörtenhuber, DoGA consortium, Juha Kere, W. Glen Pyle, Jonas Donner, Alex V. Postma, Tosso Leeb, Göran Andersson, Marjo K. Hytönen, Jens Häggström, Maria Wiberg, Jana Friederich, Jenny Eberhard, Magdalena Harakalova*, Frank G. van Steenbeek*, Gerhard Wess*, Hannes Lohi*#

* Equal contribution
# corresponding author:
Hannes Lohi, Haartmaninkatu 8, PL 63, 00014 Helsingin yliopisto, Helsinki, Finland; hannes.lohi@helsinki.fi

Summary:

Additional file 1: Fig. S1. Manhattan plots from GWAS analyses with different subcohort combinations of affected Dobermanns. **Fig. S2.** Multi-dimensional scaling (left) and quantile-quantile plots (right) from GWAS analyses with different subcohort combinations of affected Dobermanns. **Fig. S3.** Locus plots from an extended GWAS analysis with 235 cases from the “echo only”, “echo + arrhythmia” and “CHF” subcohorts and Utrecht cohort, and 143 controls from the “healthy” subcohort.

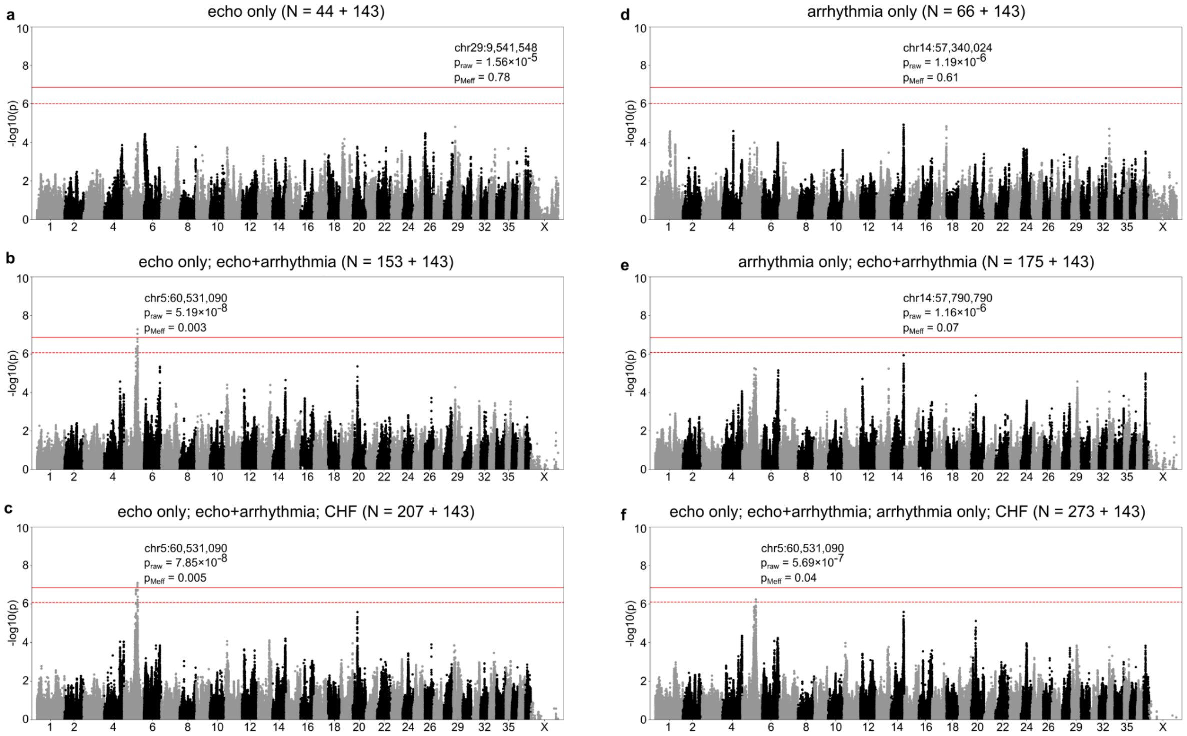

Fig. S1. Manhattan plots from GWAS analyses with different subcohort combinations of affected Dobermanns. The same 143 unaffected, at least six years old Dobermanns were utilized as controls in all analyses. The affected subcohorts included in each analysis were: a) “echo only” (affected N=44) b) “echo only” and “echo + arrhythmia” (affected N=153) c) “echo only”, “echo + arrhythmia”, and “CHF” (affected N=207) d) “arrhythmia only” (affected N=66) e) “arrhythmia only” and “echo + arrhythmia” (affected N=175) f) “echo only”, “echo + arrhythmia”, “arrhythmia only” and “CHF” (affected N=273).

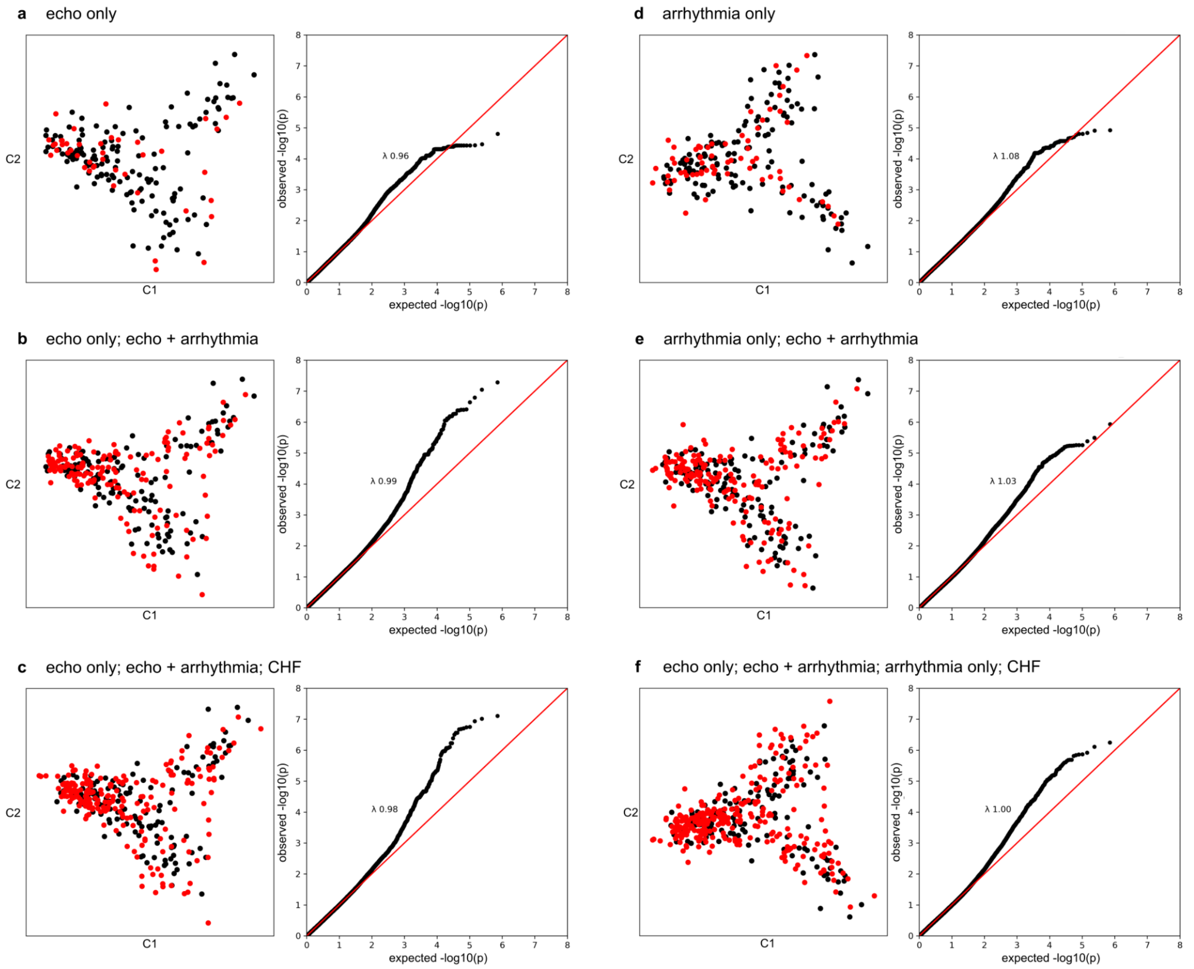

Fig. S2. Multi-dimensional scaling (left) and quantile-quantile plots (right) from GWAS analyses with different subcohort combinations of affected Dobermanns. The affected subcohorts included in each analysis were: a) “echo only” (lambda=0.96) b) “echo only” and “echo + arrhythmia” (lambda=0.99) c) “echo only”, “echo + arrhythmia”, and “CHF” (lambda=0.98) d) “arrhythmia only” (lambda=1.08) e) “arrhythmia only” and “echo + arrhythmia” (lambda=1.03) f) “echo only”, “echo + arrhythmia”, “arrhythmia only” and “CHF” (lambda=1.00).

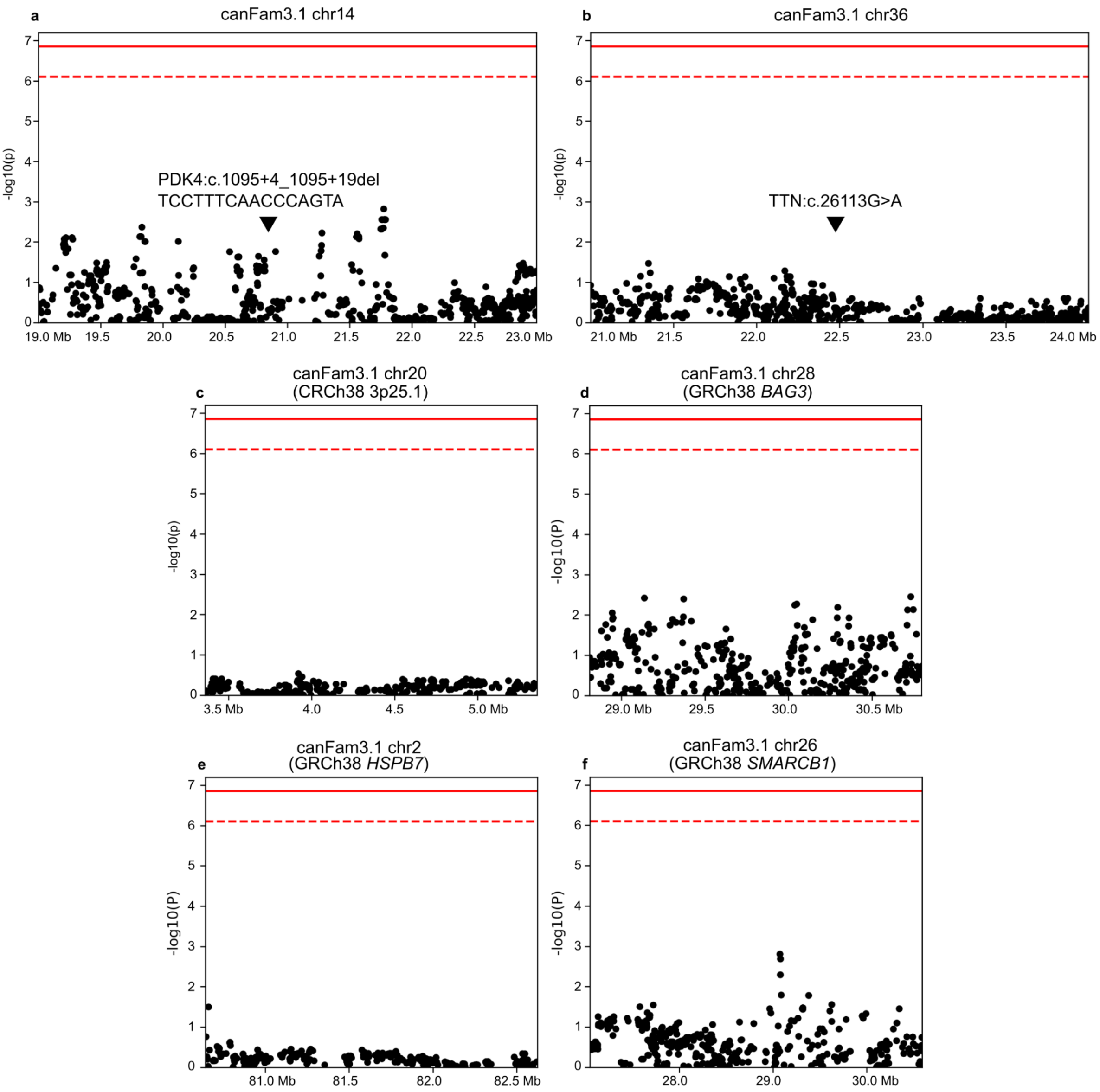

Fig. S3. Locus plots from an extended GWAS analysis with 235 cases from the “echo only”, “echo + arrhythmia” and “CHF” subcohorts and Utrecht cohort, and 143 controls from the “healthy” subcohort. Bonferroni-corrected and Meff significance thresholds are indicated with solid and dashed red lines, respectively. a) Region at chr14:19.0-23.0 Mb. The location of the previously DCM-linked PDK4 variant is indicated with a black triangle. b) Region at chr36:21.0-24.0 Mb. The location of the previously DCM-linked TTN variant is indicated with a black triangle. c) Region at chr20:3.4-5.4 Mb syntenic to a human DCM locus at GRCh38 3p25.1. d) Region at chr28:28.8-30.8 Mb syntenic to a human DCM locus at GRCh38 BAG3. e) Region at chr2:80.6-82.6 Mb syntenic to a human DCM locus at GRCh38 HSPB7. f) Region at 26:28.6-28.8 Mb syntenic to a human DCM locus at GRCh38 chr22q11.23 (SMARCB1).
